# Clinical Blood Metabogram: Application to Overweight and Obese Patients

**DOI:** 10.3390/metabo13070798

**Published:** 2023-06-27

**Authors:** Petr G. Lokhov, Elena E. Balashova, Oxana P. Trifonova, Dmitry L. Maslov, Oksana A. Plotnikova, Khaider K. Sharafetdinov, Dmitry B. Nikityuk, Victor A. Tutelyan, Elena A. Ponomarenko, Alexander I. Archakov

**Affiliations:** 1 Institute of Biomedical Chemistry, 10 Building 8, Pogodinskaya Street, 119121 Moscow, Russia; balashlen@mail.ru (E.E.B.); oxana.trifonova@gmail.com (O.P.T.); dlmaslov@mail.ru (D.L.M.); elena.ponomarenko@ibmc.msk.ru (E.A.P.); alexander.archakov@ibmc.msk.ru (A.I.A.); 2 Federal Research Centre of Nutrition, Biotechnology and Food Safety, Russian Academy of Sciences, Ustinsky Passage 2/14, 109240 Moscow, Russia; plotnikova@ion.ru (O.A.P.); sharafetdinov@ion.ru (K.K.S.); nikitjuk@ion.ru (D.B.N.); tutelyan@ion.ru (V.A.T.)

**Keywords:** metabogram, metabolomics, blood, diagnostics, mass spectrometry, clinical blood tests, personalized metabolomics

## Abstract

Recently, the concept of a mass spectrometric blood metabogram was introduced, which allows the analysis of the blood metabolome in terms of the time, cost, and reproducibility of clinical laboratory tests. It was demonstrated that the components of the metabogram are related groups of the blood metabolites associated with humoral regulation; the metabolism of lipids, carbohydrates, and amines; lipid intake into the organism; and liver function, thereby providing clinically relevant information. The purpose of this work was to evaluate the relevance of using the metabogram in a disease. To do this, the metabogram was used to analyze patients with various degrees of metabolic alterations associated with obesity. The study involved 20 healthy individuals, 20 overweight individuals, and 60 individuals with class 1, 2, or 3 obesity. The results showed that the metabogram revealed obesity-associated metabolic alterations, including changes in the blood levels of steroids, amino acids, fatty acids, and phospholipids, which are consistent with the available scientific data to date. Therefore, the metabogram allows testing of metabolically unhealthy overweight or obese patients, providing both a general overview of their metabolic alterations and detailing their individual characteristics. It was concluded that the metabogram is an accurate and clinically applicable test for assessing an individual’s metabolic status in disease.

## 1. Introduction

The advent of high-throughput analytical methods, which are used in omics sciences, notably metabolomics, is a predicted trend in the development of clinical laboratory tests [1,2]. However, there might be differences in how metabolomics technologies can be applied in clinics. A single-subject (N-of-1) study using omics technologies to assess a person’s biomaterial is the logical strategy. The multi-omics strategy, which uses genomics, transcriptomics, proteomics, and metabolomics methods to examine the biomaterial of a single individual in an N-of-1 study, is one of the most widely used [3,4,5,6]. In 2012, the Integrated Personal Omics Profiling (iPOP) project was initiated [7], where different omics data combined with a set of participant parameters (stress levels, diet, activity, medical history) are used to characterize an organism’s normal state [8,9,10]. The 100 K person wellness project was proposed in 2014, and the Arivale program supported it in 2015 [11]. In this project, the information collected over time for each participant, including genome, metabolome, microbiome, and digital self-measurement data, was used to provide recommendations for improving wellness and avoiding disease. In 2017, the Pioneer 100 Wellness Project (P100) started, which is based on data from genome sequences, clinical tests, metabolomes, proteomes, and microbiomes, as well as frequent activity measurements for individuals [12]. Despite the fact that the multi-omics approach is an efficient way to collect personal molecular data through the application of several omics technologies [13,14,15], its implementation in medicine is complicated and therefore slow. The multi-omics initiatives point out that further standardization of methods and improved quality control are needed to increase the reproducibility and reliability of multi-omics data [16].

Apart from other omics tests, single-subject (N-of-1) metabolomics studies are also being introduced into clinical practice, usually in the form of laboratory-developed tests (LDTs). An LDT is a subset of in vitro diagnostic (IVD) devices [17,18,19,20] defined by the US Food and Drug Administration (FDA) as “in vitro diagnostic tests that are manufactured and used in a single laboratory”. The LDT format avoids the difficulty of metabolomics implementation in clinical laboratories by regulating metabolomics test implementation by the protocols and standardization acts of only one laboratory [21]. In 2018, Metabolon Inc. developed several LDTs (e.g., Meta UDx™, Meta IMD™, and Meta IMD™Plus) intended to detect deviations in major metabolic pathways, biomarkers unmeasurable in other ways, and the diagnosis of some genetic disorders. Another one, Nightingale Health, uses a CE-marked IVD device and offers to estimate the “age you are likely to live before falling ill from any of the top 10 diseases” from a single finger prick blood sample [22]. Using blood NMR spectroscopy, the service estimates healthy years based on previously collected data on hundreds of thousands of people. Thus, the implementation of metabolomics tests in the LDT format is one of the options for facilitating the introduction of metabolomics into medicine.

The implementation of a personal metabolomics study can also be facilitated by measuring a small subset of metabolites. The Ajinomoto Group’s AminoIndex^®^ Cancer Screening (AICS^®^) offers minimally invasive, early cancer screening through the analysis of plasma amino acids using LC-MS [23]. Due to the measurement of only a small subset of metabolites, the AminoIndex^®^ service is an example of how metabolomics has been successfully applied in clinical settings thanks to its simplification.

**Figure 1:**
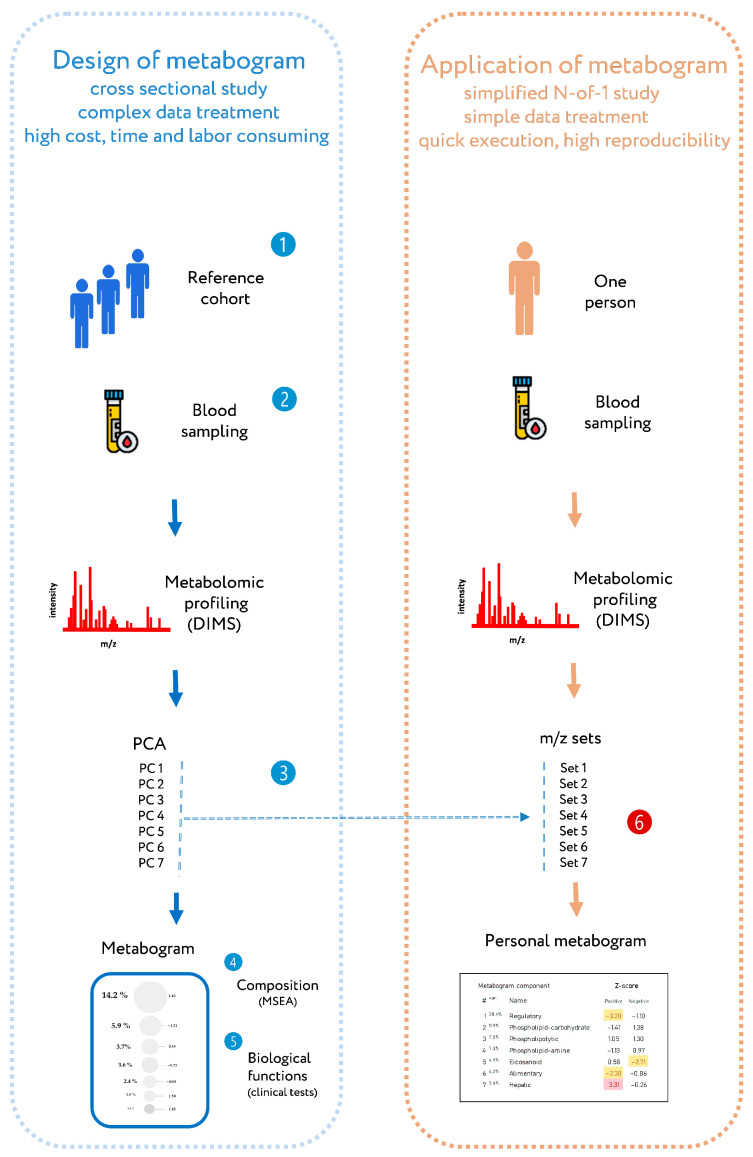
Workflow for designing a metabogram and its application as a clinical lab test. To design a metabogram, blood plasma samples are taken from healthy people (**1**), and after sample preparation, the mass spectra of blood metabolites are obtained by direct infusion mass spectrometry (DIMS) (**2**). The resulting mass peak lists are analyzed by principal component analysis (PCA) to identify the mass peak groups to form metabogram components (**3**). To characterize metabogram components, their composition was determined by identifying the chemical substances, with which they are enriched (by metabolite set enrichment analysis, MSEA) (**4**). Metabogram components are compared with clinical blood tests to reveal their functional characteristics (**5**). The design of the metabogram according to this workflow was conducted in previous work [24]. For routine application of the designed metabogram as a fast clinical test, characterized sets of mass spectrometry peaks corresponding to metabogram components are used (**6**). This workflow for applying the metabogram was used in this study and is the prototype for the clinical use of the metabogram as a laboratory-developed test (LDT). Color coding: red indicates upregulation in the corresponding metabogram component; yellow indicates downregulation in the corresponding metabogram component.

Recently, a new personalized metabolomics method called the metabogram has been introduced [24], which is also a simplified N-out-1 metabolomics study. The blood metabogram was designed using well-known methods such as direct infusion mass spectrometry (DIMS), principal component analysis (PCA), and metabolite set enrichment analysis (MSEA). The metabogram avoids the complexity of every N-of-1 metabolomics study and is characterized by quick execution, high reproducibility, simple data processing, and straightforward results interpretation, which should facilitate its future implementation in the clinic in the LDT format (Figure 1).

The metabogram shows a variety of clinically relevant information from the blood metabolome groups involving humoral regulation, lipid–carbohydrate and lipid–amine metabolism, eicosanoids, amino acids, lipid intake into the organism, and liver function. The confirmation of the metabogram’s clinical potential is the major purpose of this work. To achieve this, patients with diverse degrees of metabolic dysfunction linked to various stages of obesity are studied using the metabogram.

## 2. Materials and Methods

### 2.1. Subjects

Healthy, overweight, and obese volunteers (n = 100) were examined by the medical board at the Federal State Budgetary Institution “Nutrition and Biotechnology” (Moscow, Russia). The groups of cases included volunteers with obesity of varying stages with a diagnosis of E 66.0, according to the International Classification of Diseases (obesity of exchange-alimentary origin). Subject selection, blood sampling, and mass spectrometry analysis were conducted within the frame of a previous metabolomics study conducted in 2020 [25] and supported by the Program of the Presidium of the Russian Academy of Sciences (“Proteomic and Metabolomic Profile of Healthy Human”).

### 2.2. Mass Spectrometry Analysis of Blood Samples

Venous blood sampling, sample preparation, mass spectrometry analysis, mass spectra processing, and mass list processing (alignment, standardization, and normalization) were conducted as described previously on the same equipment and with the same materials [25]. Aligned and standardized mass lists are presented in Table S2.

### 2.3. Design of Metabogram

The design of the metabogram using a reference cohort of healthy subjects was conducted in previous work, and the details of this are described in [24]. Briefly, to design the metabogram, blood plasma samples of healthy men were analyzed using DIMS (Figure 1). After data preprocessing (alignment, standardization, and normalization), the resulting lists of mass peaks were analyzed using PCA. The sets of mass peaks corresponding to the highest positive or lowest negative principal component coefficients (loadings) formed the blood metabolome components (BMCs). The first seven BMCs, explaining approximately 70% of blood metabolome variance, formed the metabogram components. Applying MSEA, the composition of metabogram components was determined by identifying the chemical classes with which they are enriched (Table 1). To clarify the biological specificity of the metabogram components, clinical blood tests were used. Due to the fact that the principal components have positive and negative coefficients (loadings) involved in the formation of the metabogram components, each metabogram component has two Z-score scales reflecting their measure, called “positive” and “negative” parts, respectively. Z-scores of the metabogram components from −1.64 to +1.64 are in the normal range; up- and downregulation correspond to higher and lower Z-score values, respectively.

The components of the metabogram are functionally related groups of the blood metabolites associated with humoral regulation (component 1 called “regulatory”), lipid–carbohydrate metabolism (component 2), phospholypolysis (component 3 called “phospholipolytic”), lipid–amine metabolism (component 4), the level of different metabolites including oxidized fatty acids (component 5 called “eicosanoid”), lipid intake into the organism (component 6 called “alimentary”), and liver function (component 7 called “hepatic”), thereby providing clinically relevant information.

### 2.4. Personal Metabograms

Personal metabograms, which are in fact the prototype of the clinical laboratory test, were obtained using the study cohort (see Section 2.1), which consisted of subjects with normal, overweight, and obese bodies. The mass lists were standardized, normalized, and then aligned with the *m*/*z* values of the metabogram (i.e., with seven *m*/*z* sets corresponding to seven metabogram components) developed using the reference cohort (see Section 2.3). Then, the Z-scores for the metabogram components, reflecting the increase or decrease in the concentration of metabolites comprising them, were calculated using the mass peak intensities (by averaging the Z-scores for peaks belonging to the same metabogram component) [24].

### 2.5. Statistical Analysis

#### 2.5.1. Cluster Analysis

To overview metabogram types demonstrating deviations in the blood metabolome in overweight and obesity, a cluster analysis was carried out. For this, Euclidian distances between metabograms (Z-scores) were calculated using the *pdist* function (Matlab). An agglomerative hierarchical cluster tree was generated by the *linkage* function using the “ward” algorithm for computing the distance between clusters. The *dendrogram* function was used to plot the dendrogram.

#### 2.5.2. Correlation Analysis

The connection between metabogram components and organismal parameters in obesity was revealed by a correlation analysis. Spearman’s correlation between the Z-scores of the metabogram components showing their up- and downregulation and the clinical test results for each person was calculated using the *corr* function of the Matlab program. The correlation between the metabogram components themselves was also calculated to identify the relationship between them in obesity.

#### 2.5.3. Diagnostic Parameters

To assess the diagnostic potential of the metabogram for overweight and obese patients, the following diagnostic parameters were evaluated: sensitivity—the percentage of correctly identified positive results (the illness is correctly assigned to overweight and obese patients); specificity—the percentage of correctly identified negative results (the illness is correctly not assigned to control patients); and accuracy—the percentage of correctly identified positive and negative results. Diagnostic parameters were measured across aligned cohorts as well as using sex-stratified cohorts.

## 3. Results

### 3.1. Studied Subjects

One hundred volunteers—twenty healthy, twenty overweight, and sixty with class 1, 2, or 3 obesity—were selected for the study. Table 2 summarizes the cohort characteristics. An anamnesis was collected, the overall condition of the body was evaluated by a doctor, a laboratory study of blood and urine was carried out, resting energy expenditures were determined, and the body composition was estimated by bioimpedance measurements (Table S1). Based on the results obtained, the doctor categorized the volunteers into the appropriate group. The presence of hyperuricemia, dyslipidemia, and steatosis (in class 3 obesity) in the case groups was allowed.

### 3.2. Metabogram Data

**Figure 2:**
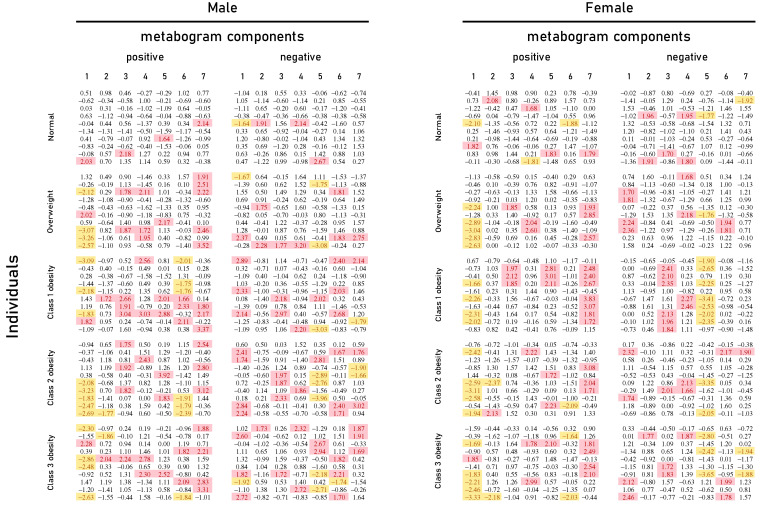
Metabogram data for normal (control), overweight, and obese patients. Z-score values are presented, which are a measure of the metabogram component (from −1.64 to +1.64 is the normal range; up- and downregulation correspond to higher and lower Z-score values, respectively). Color coding: red indicates upregulation in the corresponding metabogram component; yellow indicates downregulation in the corresponding metabogram component.

Mass spectrometry of blood plasma generated typical mass spectra of the low molecular weight fraction of blood. Up to about *m/z* 600, peaks of metabolites of various classes were observed, and above *m/z* 600, intense peaks of various phospholipids were observed. On average, 9333 peaks were detected in the spectrum. These mass spectrometry data were used to obtain personal metabograms for all subjects participating in the study (Figure 2).

**Figure 3:**
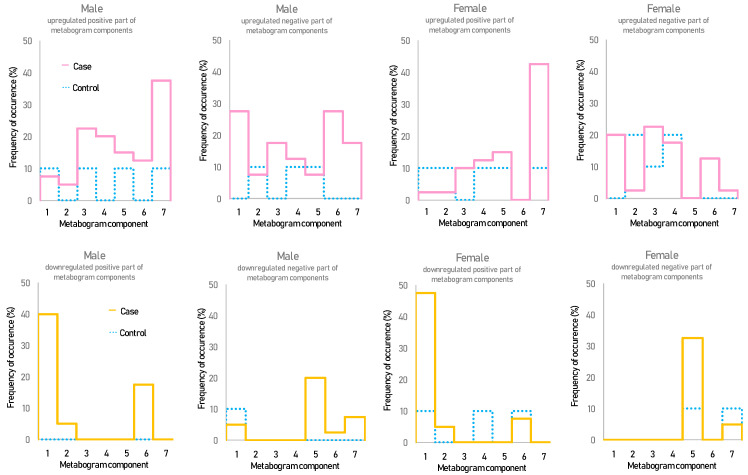
The frequency of deviations from the norm in the blood metabogram components for overweight and obese patients. The metabogram component deviates from the norm if its Z-score is less than −1.64 (the metabolites composing the metabogram component are downregulated) or above +1.64 (the metabolites composing the metabogram component are upregulated).

Figure 2 shows that overweight and obese patients have deviations in their metabogram components more frequently than control individuals. Several components deviate from the norm more often. For example, the most frequent is the downregulation of the positive components 1 and 6, the negative component 5, and the upregulation of the positive component 7 (Figure 3).

### 3.3. Statistical Data and Diagnostic Parameters

Table 3 presents the results of the *t*-test showing the significance of differences for the components of the metabogram in the case–control comparison. Additionally, in this table, diagnostic parameters for metabogram components are presented, allowing judgment of their diagnostic potential. The positive components 1 and 7 show the highest statistical significance in the case–control comparison and the highest diagnostic performance.

Table 4 displays diagnostic parameters calculated based on divergence from the normal range of any metabogram component (i.e., based on the detection of any metabolic alteration regardless of the number of metabogram components). The data in the table on the sensitivity and specificity of diagnostics show the possibility of detecting obesity-related metabolic alterations in both overweight and obese men and women.

### 3.4. Relationship between Metabogram Components

**Figure 4:**
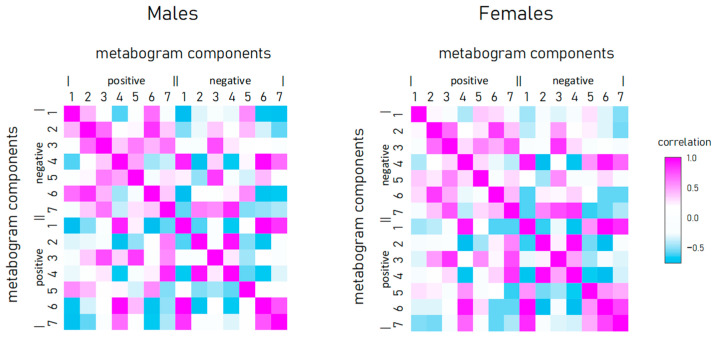
Correlation of metabogram components with each other and calculated for normal, overweight, and class 1–3 obesity patients.

To reveal the connection between metabogram components in obese individuals, the correlation between them was calculated (Figure 4). Connection strength varies widely from strong positive (e.g., between positive components 2 and 3) to strong negative (e.g., between positive component 1 and negative 7), and has approximately the same pattern for men and women.

**Figure 5:**
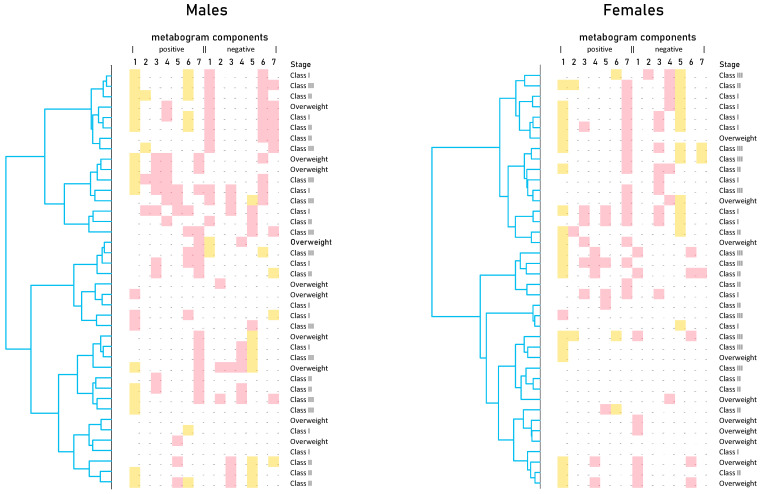
Dendrograms of blood metabograms of overweight and obese patients involved in the study. Color coding: red indicates upregulation in the corresponding metabogram component; yellow indicates downregulation in the corresponding metabogram component.

Cluster analysis was used to identify patterns formed by metabogram components (Figure 5). Clustering by stage of obesity was not revealed. Some clusters, formed by different combinations of the most often deviating metabogram components, may be considered typical for overweight and obese patients. See, for example, the upper clusters on the dendrograms for males and females (Figure 5).

**Figure 6:**
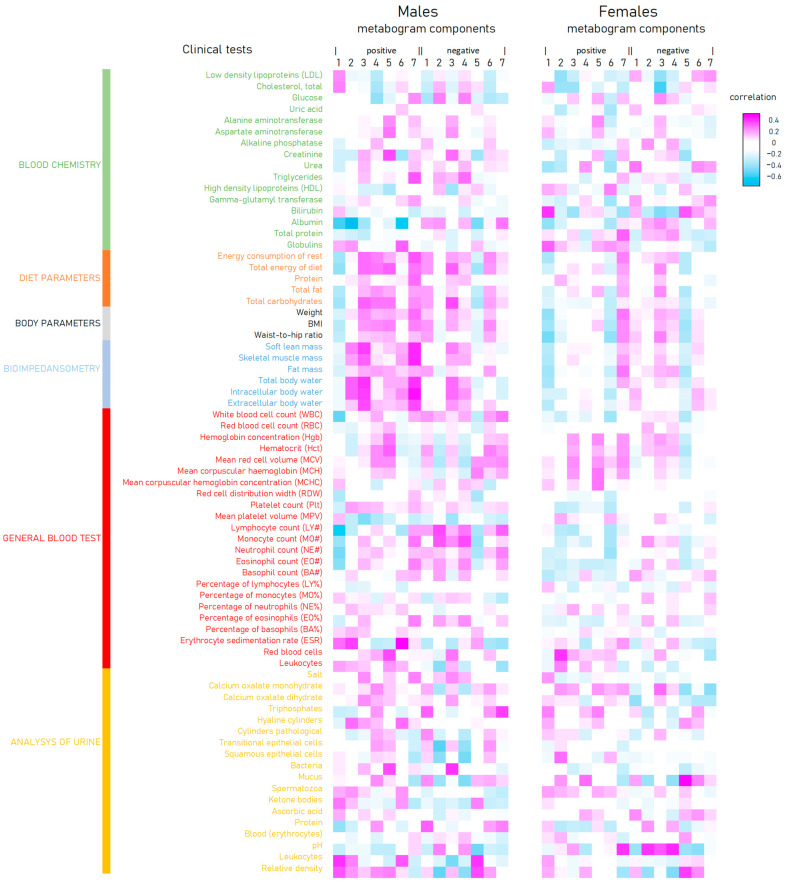
Correlation of clinical tests and body parameters with metabogram components calculated for normal (control), overweight, and class 1–3 obesity patients.

The correlation of the metabogram components with clinical tests is presented in Figure 6. There is an inverse correlation between the positive 1 and 6 and negative 5 components and diet, body, and bioimpendasometry data. A positive correlation for these tests is observed with the positive component 7 of the metabogram. This fact is in full accordance with the deviation in these components for overweight and obesity, as shown in Figure 3.

### 3.5. Metabogram Types in Obesity

**Figure 7:**
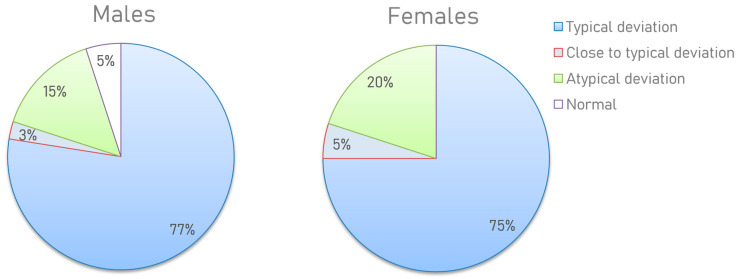
Distribution of the metabogram types in overweight and obese men and women. The positive 1, positive 6, and negative 5 components (in one of them or their various combinations) have been downregulated or the positive 7 has been upregulated in a metabogram with a “typical deviation”. If the metabogram component(s) is close to the limit of the norm, the “close to a typical deviation” metabotype is detected (when retesting, a metabogram may fall into a group with a typical deviation). “Atypical metabograms” have component deviations that are not the same as those of typical metabograms.

Based on the results obtained, several types of metabograms can be distinguished. Metabograms with the most frequent deviations in the four components, observed in 76% of cases, can be considered typical for individuals with overweight and obesity (Figure 7). In total, 4% of people have metabogram values that are marginal for the norm. This suggests that the metabograms may fall outside the norm at retesting. A total of 2.5% (5% of men and 0% of women) had no abnormalities, which does not mean their complete absence since the metabogram covers only 70% of the variance in the blood metabolome.

**Figure 8:**
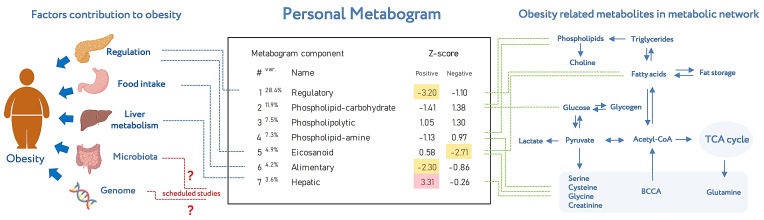
An example of metabogram. “Var” superscript shows the percentage of the variance explained by the metabogram component. The Z-score value is a measure of the metabogram component (from −1.64 to +1.64 is the normal range). “Up-“ and “downregulation” correspond to higher and lower Z-scores, respectively. The frequently deviated components of the metabogram in overweight and obese patients are selected by color (red indicates upregulation in the corresponding metabogram component; yellow indicates downregulation in the corresponding metabogram component). To the left of the metabogram are the factors contributing to the development of obesity (adapted from [26]) and where they are mainly reflected in the metabogram. The reflection of the microbiota and genome in the components of the metabogram (indicated by the sign ‘?’) will be established in future studies. To the right of the metabogram is the relationship of obesity-related metabolites in the metabolic network (adapted from [27]) and in which metabogram components they may be reflected.

Metabogram displays personal metabolic data which can be presented in various formats (as a table or diagram). Figure 8 shows the metabogram in a simple format, which shows the number of the metabogram component, the variance in the blood metabolome explained by the component, the name given to the component, and the values of the component on the Z scale. The figure also provides an overview of the factors contributing to obesity developments and obesity-related metabolites in the metabolic network reflected in the metabogram components.

## 4. Discussion

The concept of the metabogram is to introduce metabolomics into the clinic by simplifying the single-subject metabolomics study. The metabogram approach makes it possible to dispose of the identification and analysis of individual metabolites, of which there are thousands in biological samples, making any metabolomics study complex, time-consuming, and expensive [24]. For this, in the metabogram, only groups of related metabolites are processed, and the application of MSEA [28] quickly estimates the enrichment of these groups with metabolite classes. Thus, the complex identification of individual metabolites is replaced by group analysis using this well-known and fast method. Moreover, averaging metabolite data (peak intensities) within one group leads to increased data reproducibility. The coefficient of variation (CV) for metabogram components is as low as 1.8% [24], which is not achievable for most individual metabolites [29]. In this study, individuals with varied metabolic alterations related to overweight and obesity were assessed using a metabogram in order to validate the clinical value of the metabogram.

Obesity is an adaptation of the body to additional energy intake and reduced energy expenditure. Obesity plays a crucial role in the development of metabolic syndrome, insulin resistance, dyslipidemia, arterial hypertension, type 2 diabetes, and an increased risk of cardiovascular disease [30,31,32,33,34]. Additionally, obese individuals are more likely to suffer from cancer, asthma, gallbladder disease, osteoarthritis, and chronic pain [35]. It can be argued that the metabolic features of obese patients have long been under the scrutiny of researchers and, in many ways, are already well described, which makes this pathology attractive for metabogram testing.

The clinical manifestations of overweight or obesity are heterogeneous. In contrast to metabolically healthy obesity (MHO), metabolically unhealthy obesity (MUO) has unfavorable metabolic profiles characterized by low insulin sensitivity, abnormal blood pressure, and unfavorable lipid, inflammation, hormone, liver enzyme, and immune profiles [36]. The blood metabolome abnormalities in such MUO people may be responsible for the comparatively high level of diagnostic specificity, sensitivity, and accuracy of the metabogram demonstrated in the study (Table 4).

One of the major groups in the metabolic signature of obesity is steroids [37]. For example, testosterone has been described as antiadipogenic; its administration in adult men reduces abdominal fat by stimulating lipolysis and thereby reducing fat accumulation in adipocytes [38]. A strong inverse correlation was found between testosterone levels and obesity in overweight adult men [39,40,41]. In women, however, the data are less uniform, ranging from no association [40] or a positive correlation observed in overweight and obese women [39,42] to an inverse correlation [43,44]. The metabogram data are consistent with scientific evidence on the relationship between steroids and obesity. The first metabogram component, named regulatory due to its enrichment with steroids (Table 1), is downregulated in people with overweight and obesity. Moreover, the metabogram brings clarity for women; there is a clear decrease in steroid levels, at least in women in the fertile period involved in the study (Figure 2 and Figure 3).

The second major group of metabolites dysregulated in obesity is amino acids, particularly branched-chain amino acids and aromatic amino acids [37]. For example, phenylalanine concentrations are higher in obese individuals [45,46,47]. Tyrosine levels, a hydroxylation product of phenylalanine metabolism, are associated with an increase in the hepatic fat content [48]. The metabogram has several components related to amino acids (Table 1). A small part that positively correlates with phospholipids is represented in negative component 4. The main part of amino acids is in the positive part of components 5 and 7, which in both women and men are either normal or upregulated and never downregulated (Figure 2 and Figure 3). Component 7 is upregulated more frequently. Thus, the metabogram is completely consistent with amino acid dysregulation in obese patients.

The next group altered in obesity is lipids other than steroids. Many studies have reported different results, namely changes in saturated and unsaturated fatty acids, lysophospholipids, and phospholipids. It is reported that saturated fatty acids positively correlate with the development of obesity [49,50]. There are studies demonstrating that some saturated fatty acids, especially long-chain, have a weak correlation with MUO compared with unsaturated fatty acids, except for palmitic and oleic acids [51,52,53]. Levels of both eicosapentaenoic acid and docosahexaenoic acid [52,54] increased in the MUO group, but they are associated with a lower risk of developing metabolic syndrome [55].

Saturated and unsaturated fatty acids associated with steroids are presented in component 1 of the metabogram (Table 1). Additionally, unsaturated fatty acids are presented in negative component 5, which is normal or downregulated in women and normal, downregulated, or, twice as rare, upregulated in men. Therefore, these unambiguous data for women and less unambiguous data for men are not contradicted by the present scientific facts.

Controversial scientific data are associated with lysophospholipids and phospholipids. Some studies report decreased levels of these compounds [56,57,58,59,60], while other data suggest a correlation between the levels of lysophospholipids and phosphatidylcholines in plasma in obese individuals, although the mechanisms are not yet fully understood [61,62]. Thus, these molecules are not considered critical metabolites in obesity signatures.

Phospholipids in the metabogram are represented in the negative components 2 and 4, which are either normal or occasionally elevated in patients, which does not contradict the scientific data and is consistent with their weak diagnostic significance. Lysophospholipids are presented in negative component 7, which deviates from the norm rarely and in different directions, which is also consistent with the obscurity of their behavior described in the scientific data.

Acylcarnitines, metabolites formed intracellularly from carnitine during the metabolism of fatty acids and amino acids, show differences in the levels between the MUO and MHO subjects [63,64]. However, there are no components in the metabogram that are enriched by acylcarnitines.

The agreement between the metabogram patterns (the most frequent deviations in regulatory, alimentary, and hepatic metabogram components for overweight and obese individuals; Figure 3 and Figure 6) and body parameters, diet, and bioimpedance measurements (Figure 4) is an additional contribution to confirming the validity of the metabogram as a clinical test.

Therefore, the collected scientific evidence to date supports that the metabogram is interpretable and accurate in terms of determining known abnormalities in the blood metabolites in obese patients. Moreover, the metabogram provides new diagnostic possibilities through a panoramic overview of the metabolites in the patient’s blood. So, only 2.5% of the subjects in the study had no deviations in the metabogram (Figure 7). This suggests that the identification and systematization of metabolic abnormalities in the diagnosis and treatment of obesity may now be at a new level.

From the results of this study, it is feasible to identify the benefits offered by the metabogram in overweight and obesity:

- The metabogram’s broad coverage of the metabolites enables the identification of the molecular phenotypes of patients (metabotypes), frequency distributions for particular metabotypes in overweight and obese patients, deviations in the blood metabolome associated with these metabotypes, and the most prevalent combinations of these deviations.
- The metabogram allows us to measure these deviations, rank them, and identify the most significant of them in overweight and obesity.
- The metabolite group-based approach used in the metabogram is an efficient way to retrieve and interpret data from the metabolome, which is challenging when dealing with individual metabolites.
- The classification of metabotypes, measurability, and interpretability of deviations in the blood metabolome using the metabogram make it possible to personalize the treatment of obesity.

Therefore, simplification of the N-of-1 metabolome study in the metabogram concept is not only technically beneficial for metabolomics implementation in clinical practice but also a way of efficient data extraction from the blood metabolome, which makes the metabogram a promising method of personalized metabolomics.

Regarding the weaknesses of the metabogram, the sample preparation protocol with methanol chosen to design the metabogram leads to a decrease in the detection of triglycerides [65], which would not be superfluous to detect metabolic deviations associated with obesity. Additionally, besides the fact that the used mass spectrometer was equipped with an ESI source, which is considered to be soft [66], some metabolites are susceptible to in-source fragmentation. This is especially true for phospholipids fragmenting into lysoforms and fatty acids [67], which affects the composition of metabogram components where such compounds are present. The next issue is associated with the metabolite databases that were used to design the metabogram, where redundancy in compound names exists. Together with the high combinatorial capabilities of lipids (e.g., phospholipids have a variety of fatty acid chains, resulting in many compounds with the same molecular weight), it makes the metabogram components enriched with such lipids less reliable.

As for the prospects of the development of the metabogram, before the metabogram is used in the clinic, it is necessary to define how the components of the metabogram relate to the genome and gut microbiota because these factors have a well-known impact on the blood metabolome [68]. The next prospect of the implementation of metabograms in laboratory diagnostics may be related to their combination with dried blood spot (DBS) samples [69], allowing the unaided collection of capillary blood at home. The subsequent transportation of DBS samples to the laboratory by mail can make metabogram tests convenient for customers and available almost everywhere, which is especially important given the prevalence of obesity.

## 5. Conclusions

It can be argued that the metabogram has clinical value, as demonstrated in overweight and obese individuals. The metabogram detects the metabolic signature of obesity, which is consistent with the accumulated scientific data. The metabogram adds new meaning to blood analyses in obese patients because it is an omics test, which provides both a general overview of metabolic abnormalities and details the individual character of these abnormalities. In addition, the metabogram brings clarity to the analysis of the blood of obese patients, eliminating inconsistencies in the vast amount of scientific data and making it clinically applicable. According to the metabogram, the most frequent deviations in the metabolome, corresponding to overweight and obesity, are associated with humoral regulation (40% and 48%), liver function (38% and 43%), the level of fatty acids in the blood (20% and 32%), and, to a lesser extent, with the nutritional factor (27% and 13% frequency of occurrence for men and women, respectively). The metabogram indicates not only the severity and frequency of the occurrence of these deviations but also their role in the functioning of the body, as reflected in the variance in the blood metabolome covered by the metabogram components. The individual picture of each patient is made up of deviations in certain components, which provide information about the obesity metabotype and provide grounds for personalizing its treatment. Thus, the design of the metabogram as a rapid clinical test, together with its demonstrated clinical relevance, justifies further efforts to introduce it into clinical practice.

## Figures and Tables

**Table 1:** Composition of the clinical blood metabogram components. Adapted from [24].

Metabolite Group	Metabogram Component ^1^						
	1 ^2^	2	3	4	5	6	7
Phosphatidylcholines		●		●			
Phosphatidylethanolamines		●		●			
Monosaccharides		●					
Saturated Fatty Acids	●						
C18 steroids	●						
C10 isoprenoids	●						
C24 bile acids						●	
Dicarboxylic acids	●				●		
Unsaturated Fatty Acids	●				●		
Lysophosphatidylcholines					●		●
Lysophosphatidylethanolamines							●
Diacylglycerols						●	●
Retinoids					●	●	
Amino acids				●	●		●
Androstane steroids	●						●
C19 steroids	●						●
Glycerophosphoglycerophosphates							●
Estrane steroids	●						
Leukotrienes					●		
Prostaglandins					●		

^1^ The blue color corresponds to the positive part of the metabogram components; the red color corresponds to the negative. ^2^ Names of metabogram components: 1—regulatory; 2—lipid–carbohydrate; 3—phospholipolytic; 4—lipid–amine; 5—eicosanoid; 6—alimentary; 7—hepatic.

**Table 2:** Study cohort characteristics.

Group	Body Height ^1^ (cm)	Body Weight (kg)	Age (Years)	Body Mass Index (kg/m^2^)	Gender (Male/Female)
Normal	173.5 ± 8.2	66.9 ± 9.4	31.3 ± 5.5	22.1 ± 1.9	10/10
Overweight	172.1 ± 12.2	82.0 ± 13.1	32.9 ± 6.7	27.5 ± 1.3	10/10
Class 1 obesity	170.6 ± 11.7	95.1 ± 13.7	29.7 ± 8.0	32.5 ± 11.7	10/10
Class 2 obesity	171.5 ± 9.4	109.1 ± 13.8	32.8 ± 8.1	36.9 ± 1.3	10/10
Class 3 obesity	172.3 ± 9.9	141.0 ± 27.4	34.5 ± 6.5	47.3 ± 6.1	10/10

^1^ Mean ± standard deviation.

**Table 3:** Diagnostic parameters of the metabogram components for the detection of overweight and class 1–3 obesity stages.

Metabogram Component	*t*-Test (*p*-Value)	Diagnostic Parameters (%)		
		Sensitivity	Specificity	Accuracy
Positive parts of metabogram components				
1	0.0007	49	85	56
2	0.504	9	95	26
3	0.024	16	95	32
4	0.143	16	90	31
5	0.011	15	90	30
6	0.204	19	95	34
7	0.002	40	90	50
All (1–7)	0.246	83	45	75
Negative parts of metabogram components				
1	0.093	27	95	40
2	0.851	5	85	21
3	0.010	20	95	35
4	0.102	15	85	29
5	0.023	31	90	43
6	0.122	21	100	37
7	0.515	16	95	32
All (1–7)	0.062	70	70	70

**Table 4:** Diagnostic parameters for the detection of obesity by metabogram.

Groups (Cases Versus Controls)	Metabogram Components ^1^								
	Positive ^2^			Negative			Both		
	Sensitivity (%)	Specificity (%)	Accuracy (%)	Sensitivity (%)	Specificity (%)	Accuracy (%)	Sensitivity (%)	Specificity (%)	Accuracy (%)
Overweight ^males^ versus Normal ^males 3^	80	60	70	60	80	70	90	60	50
Overweight ^males^ versus Normal ^all^	80	55	60	60	70	65	90	45	60
Overweight ^females^ versus Normal ^females^	60	50	55	60	60	60	90	30	60
Overweight ^females^ versus Normal ^all^	60	55	57	60	70	67	90	30	50
Overweight ^all^ versus Normal ^all^	70	55	63	60	80	70	90	45	68
Class 1 obesity ^males^ versus Normal ^males^	80	60	70	60	80	70	80	60	70
Class 2 obesity ^males^ versus Normal ^males^	90	60	75	90	80	85	100	60	80
Class 3 obesity ^males^ versus Normal ^males^	100	60	80	90	80	85	100	60	80
Class 1 obesity ^males^ versus Normal ^all^	80	55	60	60	70	67	80	45	57
Class 2 obesity ^males^ versus Normal ^all^	90	55	67	90	70	77	100	45	63
Class 3 obesity ^males^ versus Normal ^all^	100	55	70	90	70	77	100	45	63
Class 1 obesity ^females^ versus Normal ^females^	70	50	60	100	60	80	90	30	60
Class 2 obesity ^females^ versus Normal ^females^	80	50	65	50	60	55	80	30	55
Class 3 obesity ^females^ versus Normal ^females^	90	50	70	60	60	60	90	30	60
Class 1 obesity ^females^ versus Normal ^all^	70	55	60	100	70	85	90	45	60
Class 2 obesity ^females^ versus Normal ^all^	80	55	63	50	70	63	80	45	57
Class 3 obesity ^females^ versus Normal ^all^	90	55	67	60	70	65	90	45	68

^1^ All seven components of the metabogram were involved in the calculation. ^2^ “Positive” and “negative” correspond to two parts of Z-score scales reflecting component measures (see Section 2.3). ^3^ The superscript corresponds to the group used (“males”: only males; “females”: only females; “all”: males and females together).

## Data Availability

The data presented in this study are available as Supplementary Materials and on request from the corresponding author due to privacy.

## Supplementary Materials

The following are available online at https://www.mdpi.com/article/10.3390/metabo13070798/s1, Table S1: Clinical test results for studied subjects. Table S2: Aligned and standardized mass lists.
